# Effects of prostaglandin E_1_ nebulization of ventilated lung under 60%O_2_ one lung ventilation on patients’ oxygenation and oxidative stress: a randomised controlled trial

**DOI:** 10.1186/s12931-020-01380-6

**Published:** 2020-05-13

**Authors:** Pengyi Li, Lianbing Gu, Qingming Bian, Jing Tan, Dian Jiao, Fei Wu, Zeping Xu, Lijun Wang

**Affiliations:** 1 Department of Anesthesiology, Jiangsu Cancer Hospital & Jiangsu Institute of Cancer Research & The Affiliated Cancer Hospital of Nanjing Medical University, No. 42 Baiziting, Xuanwu District, Nanjing, 210009 China; 2 Renji Clinical School, Shanghai Jiao Tong University School of Medicine, Shanghai, 200000 China

**Keywords:** Prostaglandin E_1_, One-lung ventilation, Low FiO_2_

## Abstract

**Background:**

High FiO_2_ during one-lung ventilation (OLV) can improve oxygenation, but increase the risk of atelectasis and oxidative stress. The aim of this study was to analyze whether Prostaglandin E_1_ (PGE_1_) can improve oxygenation and attenuate oxidative stress during OLV under a lower FiO_2_.

**Method:**

Ninety patients selectively undergoing thoracotomy for esophageal cancer were randomly divided into three groups (*n* = 30/group): Group P (FiO_2_ = 0.6, inhaling PGE_1_ 0.1 μg/kg), Group L (FiO_2_ = 0.6) and Group C (FiO_2_ = 1.0). The primary outcomes were oxygenation and pulmonary shunt during OLV. Secondary outcomes included haemodynamics, respiratory mechanics and oxidative stress in serum.

**Results:**

Patients in Group P had significantly higher PaO_2_ and lower shunt fraction in 30 min of OLV compared with Group L. Compared with Group C, patients in Group P had similar levels of PaO_2_/FiO_2_ in 60 min and higher levels of PaO_2_/FiO_2_ at 2 h during OLV. The levels of PvO_2_ and SvO_2_ in Group P and Group L were significantly lower than Group C. Patients in Group P and Group L had significantly higher levels of superoxide dismutase and lower levels of malondialdehyde than Group C. No significant differences were found in SPO_2_, ETCO_2_, PaCO_2_, Paw, HR and MAP among the three groups. The complications in Group C were significantly higher than another two groups.

**Conclusion:**

PGE_1_ can maintain adequate oxygenation in patients with low FiO_2_ (0.6) during OLV. Reducing FiO_2_ to 0.6 during OLV can decrease the levels of oxidative stress and complications after OLV.

**Trial registration:**

chictr.org.cn identifier: ChiCTR1800017100.

## Background

Adequate resection of primary lesions and dissection of lymph nodes are important for thoracotomy in treatment of esophageal cancer [1, 2]. One lung ventilation (OLV) technology provides a maneuverable and minimally mobile surgical field. However, the complication, such as intrapulmonary shunt, can lead to a dropping partial pressure of arterial oxygen (PaO_2_) and threatening hypoxemia [3, 4]. Even though high FiO_2_ (1.0) improved oxygenation [5], subsequent atelectasis and oxidative stress significantly increased the risk of acute respiratory distress syndrome (ARDS), which were the leading cause of death after surgery [6, 7]. Studies showed that high FiO_2_, high airway pressure, and prolonged OLV were independent risk factors for ARDS [8–10]. The current protective ventilation strategy recommends use of the lowest FiO_2_ as far as possible in the presence of adequate oxygenation [11].

PGE_1_ is a selective pulmonary arterial vasodilator. Study had shown that PGE_1_ could decrease pulmonary shunts and increase PaO_2_ in a dose-dependent manner during OLV [12]. However, it remains unknown about whether PGE_1_ nebulization of ventilated lung can reduce pulmonary shunts and maintain adequate oxygenation during OLV under a lower FiO_2_. The primary objective of this research was to study the effect of preemptive PGE_1_ nebulization of ventilated lung with a lower FiO_2_ on maintaining adequate oxygenation during OLV. Our secondary objective was to study the benefits of lower FiO_2_ during OLV.

Our previous animal study showed that FiO_2_ (0.6) was safe for OLV and could make a lower lung injury and oxidative stress than FiO_2_ (1.0). Thus, FiO_2_ (0.6) was used as lower FiO_2_ during OLV in this article [13]. Previous studies showed that high FiO_2_ during OLV was associated with many complications, including lung atelectasis, lung infiltration, lung injury and ARDS [6, 7, 14–16]. Reactive oxygen species produced by cells under excessive oxygen were the main reason to this complications [17, 18]. Serum levels of malondialdehyde (MDA) and superoxide dismutase (SOD) are indirect parameters of oxidative stress. Previous studies had shown that MDA was association with lung injury and SOD could attenuate the lung injury [19, 20]. In this article, levels of MDA and SOD and complications after surgery were used to evaluate the benefits of lower FiO_2_ during OLV.

## Methods

**Fig. 1:**
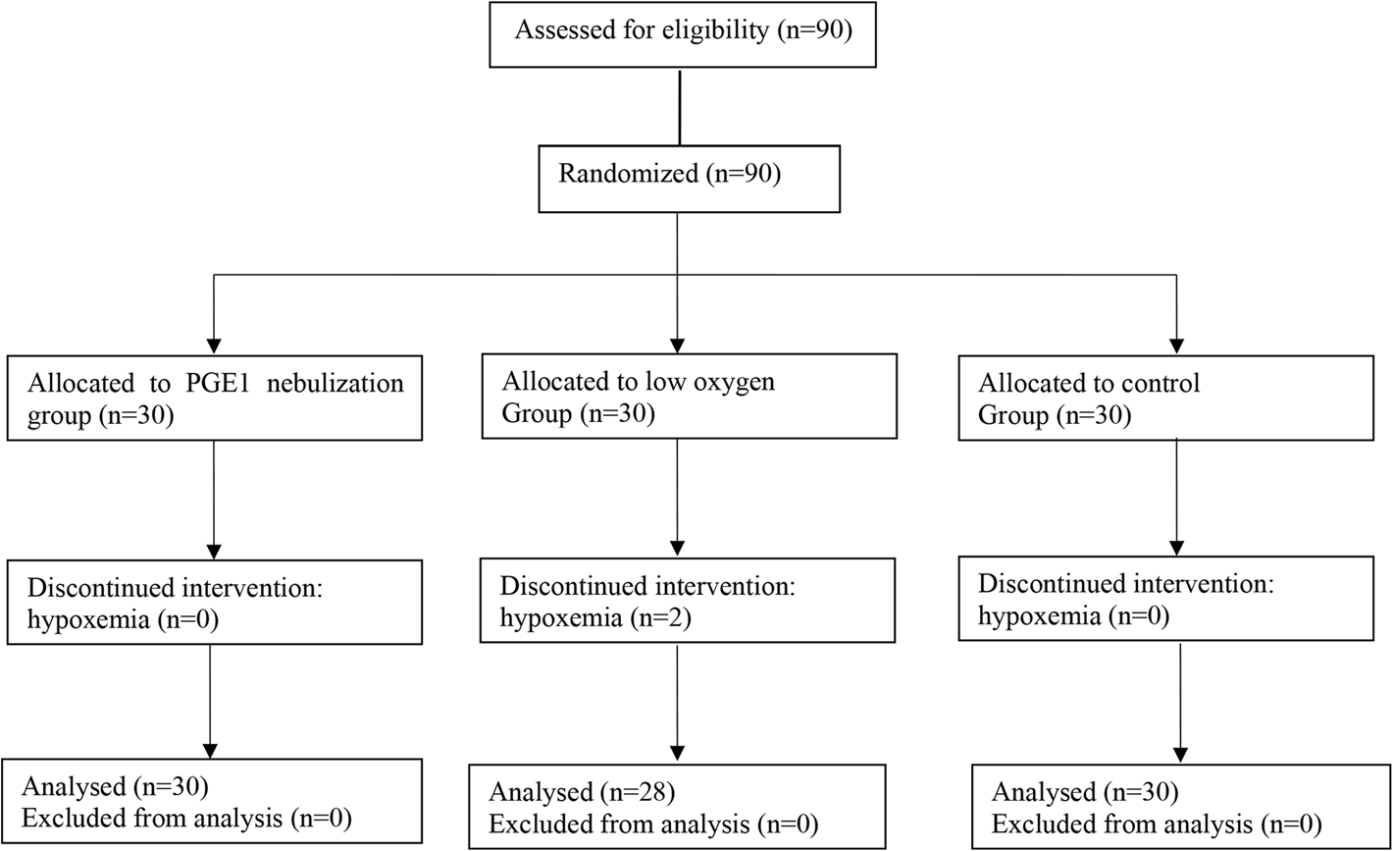
Flow diagram of study participants

Esophageal cancer patients who were scheduled for elective open radical resection were recruited at the Affiliated Cancer Hospital of Nanjing Medical University between 2015 and 2017. Esophageal cancer was diagnosed on the basis of clinical, laboratory, gastroscopy and pathology. Exclusion Criteria: (1) SpO_2_ < 90% during trial; (2) severe arrhythmia and hemodynamic instability during surgery; (3) Surgical duration more than 6 h or less than 1 h; (4) immune, endocrine, neurological and cardiovascular diseases, liver dysfunction, kidney dysfunction, glaucoma and psychiatric disorder. Patients were randomly assigned to three groups using random number table (Fig. 1): Group P (FiO_2_ = 0.6, PGE_1_ dose = 0.1 μg kg^− 1^, *n* = 30) [14], Group L (FiO_2_ = 0.6, *n* = 30) and Group C (FiO_2_ = 1.0, *n* = 30). All the participants were enrolled after written informed consent was obtained and this study was approval from the Ethics Committee of Nanjing Medical University according to the Helsinki Declaration. This randomized controlled clinical trial was registered at chictr.org.cn (identifier: ChiCTR1800017100).

### Anesthesia and intervention

All patients were under total intravenous anesthesia with left-sided double-lumen intratracheal intubation. 30 min before being sent into OR, patients were given intramuscular phenobarbital 0.1 g and atropine 0.5 mg. Upon entering the OR, central venous catheter was placed into the right internal jugular vein. Induction was done with sequential intravenous midazolam 0.05 mg kg^− 1^, fentanyl 3 ~ 4 μg kg^− 1^, propofol 1 mg kg^− 1^ and cis-atracurium 0.2 mg kg^− 1^. Left-sided double-lumen intratracheal tube was then placed, followed with confirmation of position using fiberoptic bronchoscope. Ventilation parameters were set as follows: VT = 6 ~ 8 ml/predicted body weight, RR = 12 ~ 14 /min, I: E = 1:2, ETCO_2_ = 35 ~ 45 mmHg, FiO_2_ = 0.6, PEEP = 5cmH_2_O. During OLV, theses parameters was remained unchanged and nonventilated lung was directly connected with room air. Group P and Group L had FiO_2_ of 0.6 throughout the surgery, while Group C had FiO_2_ of 1.0. Continuous intravenous pumps of remifentanil, propofol and cis-atracurium were set up for maintenance of anesthesia. Monitored intraoperative anesthesia depth and maintained bispectral index (BIS) at 40 ~ 60.

After confirming the placement of double-lumen tube with fiberoptic bronchoscope, patient was repositioned to right lateral decubitus and both lungs were ventilated. Group P was given PGE_1_ [21] (Beijing Tide Pharmaceutical Co., LTD, 10 μg/2 ml, diluted to 10 ml with normal saline) nebulization to the right lung through a reconstructed breathing circuit, while Group L and Group C were given 10 ml normal saline nebulization to the right lung, all nebulization maintained for 10 min. We modified the Yuyue 402A ultrasonic nebulizer by first sealing the bottom of nebulizer tank thus removing the air intake; and then separating the two original nebulizer outlets, so that one outlet was the new intake and the other became the only outlet towards the patient. The system is then connected to the breathing circuit [22].

### Observed parameters

An arterial catheter was placed into the radial artery, and a central venous line (two lumens 20 cm long) was introduced via the internal right jugular vein into the right atrium, and its position was confirmed by chest roentgenogram. Artery and venous blood samples were collected for blood gas analyses at post-anesthesia/pre-nebulization (T_1_), OLV 10 min (T_2_), OLV 15 min (T_3_), OLV 30 min (T_4_), OLV 60 min (T_5_), OLV 120 min (T_6_). Meanwhile, mean arterial pressure (MAP), HR and airway pressure (Paw) were recorded. Shunt fraction was calculated using this formula: Qs/Qt = (CcO_2_-CaO_2_)/(CcO_2_-CvO_2_) [23, 24].

CaO_2_ = (1.36 × hemoglobin ×SaO_2_) + (0.0031 × PaO_2_);

CvO_2_ = (1.36 × hemoglobin ×SvO_2_) + (0.0031 × PvO_2_);

CcO_2_ = ([FiO_2_ × (P_B_-P_H2O_)-PaCO_2_/Respiratory quotient] × 0.0031) + 1.36 × hemoglobin;

P_B_,760 mmHg; PH_2_O, 47 mmHg; respiratory quotient, 0.8.

### Measurement of serum malondialdehyde and superoxide dismutase

Venous blood sampling through central line was collected at T_1_, T_4_, 30 min after restarting two lung ventilation (TLV) (T_7_) and 24 h post-operation (T_8_). After centrifugation at 3000 rpm for 20 min, serum samples were frozen and stored at − 80 °C until biochemical assessment. Human MDA and SOD ELISA Kits were used to measure the concentrations of malondialdehyde (MDA) and superoxide dismutase (SOD) as manual described, respectively.

### Statistical analysis

SPSS 20.0 software (IBM Corporation, Armonk, NY, USA) was used to data analysis. At least 25 patients were required in each group to achieve a power of 0.8 and a type I error of 0.05. The data was expressed as mean ± standard deviation (SD) of at least triplicate measurements, and statistical analysis was made by t-test or ANOVA as appropriate. Counting data was tested by chi-square test. *P* < 0.05 was considered statistically significant.

## Results

A total of 90 patients (74 male, 16 female) were enrolled and randomly divided into three groups. Two patients in Group L developed hypoxemia (occurring in 10 min and 15 min during OLV, respectively) and needed to elevate FiO_2_ during OLV_._ They were excluded from Group L due to elevating FiO_2_. Table 1 showed the clinical characteristics of patients in the three groups. There were no significant differences in age, gender, BMI, ASA class, TNM class, lung function, OLV and surgical time (*p* > 0.05).

**Table 1:** Baseline characteristics of patients among three groups

	Group P (*n* = 30)	Group L (*n* = 28)	Group C (*n* = 30)	P
Age (years)	63.2 ± 6.0	63.0 ± 6.0	60.1 ± 10.6	0.111
Male, n (%)	25 (83.3%)	23 (82.1%)	24 (80%)	0.944
Body mass index (BMI)	22.6 ± 3.2	23.6 ± 6.3	23.1 ± 2.8	0.506
ASA				0.588
II	30	27	29	
III	0	1	1	
OLV duration (min)	177 ± 44	172 ± 44	171 ± 50	0.862
Surgery duration (min)	214 ± 66	206 ± 53	215 ± 46	0.778
Room air PaO_2_ (mmHg)	81 ± 9	79 ± 9	79 ± 8	0.633
Pulmonary function test				
FVC (L)	3.4 ± 0.5	3.5 ± 0.5	3.3 ± 0.5	0.322
FVC (%, predicted)	93.7 ± 5.4	94.8 ± 5.3	95.3 ± 5.3	0.531
FEV1 (L)	3.1 ± 0.3	3.2 ± 0.2	3.1 ± 0.3	0.179
FEV1 (%, predicted)	94.3 ± 5.4	95.8 ± 5.2	95.6 ± 5.2	0.525
FEV1/FVC ratio (%)	94.2 ± 7.4	93.8 ± 6.6	92.9 ± 7.1	0.761
TNM stage (n)				0.907
T1N0M0/T2N0M0/T1N1M0	17/8/2	18/7/1	17/7/3	
T2N1M0/T2N2M0/T3N0M0	1/1/1	1/1/0	1/1/1	

**Fig. 2:**
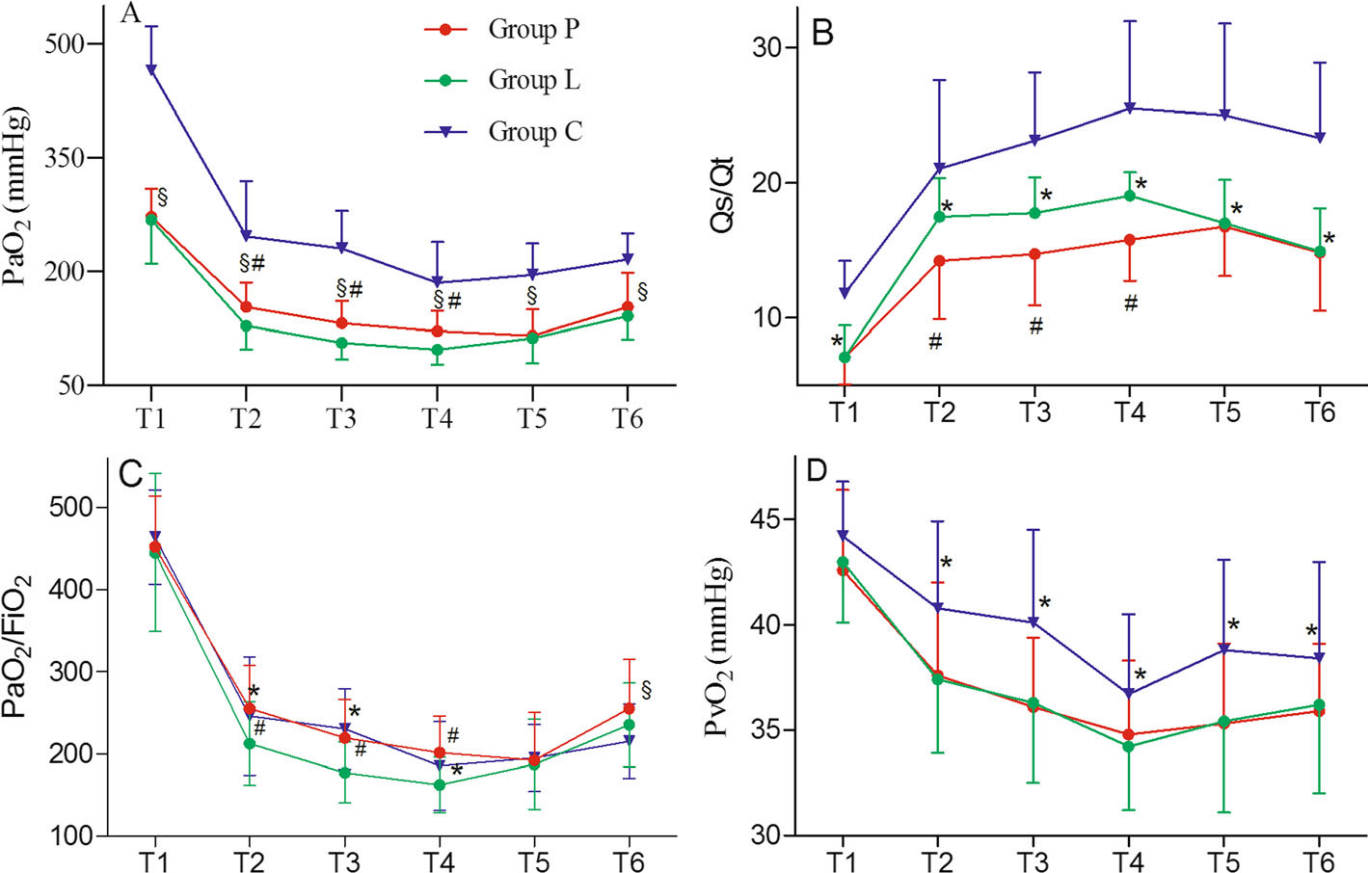
PaO_2_, Qs/Qt, PaO_2_/FiO_2_ and PvO_2_ dynamic changing during OLV in three groups. Comparison of the level of PaO_2_ (**a**), Qs/Qt (**b**), PaO_2_/FiO_2_ (**c**) and PvO_2_ (**d**) in Group P, Group L and Group C at post-anesthesia/pre-nebulization (T_1_), OLV 10 min (T_2_), OLV 15 min (T_3)_, OLV 30 min (T_4_), OLV 60 min (T_5_), OLV 120 min (T_6_). *p < 0.05 Group L vs. Group C; ^#^*p* < 0.05 Group P vs. Group L; ^§^*p* < 0.05 Group P vs. Group C

PaO_2_ and PaO_2_/FiO_2_ decreased in the first 30 min after initiation of OLV (Fig. 2a and c), while Qs/Qt increased (Fig. 2b). PaO_2,_ PaO_2_/FiO_2_ and Qs/Qt in Group L and Group C reached nadir at 30 min, while Group P did at 60 min. Group L and P had significantly lower PaO_2_ and Qs/Qt than Group C during the whole OLV. Group P had significantly higher levels of PaO_2_ than Group L, but lower levels of Qs/Qt in first 30 min during OLV (Fig. 2a and b). Group L had significantly lower PaO_2_/FiO_2_ than Group P and C in first 30 min, and no significant difference was found between Group P and Group C (Fig. 2c) in first 60 min during OLV. At OLV 120 min, Group P had significantly higher level of PaO_2_/FiO_2_ than Group C. Group C had significantly higher levels of PvO_2_ (Fig. 2d) and SvO_2_ (Table 2) than Group P and L during OLV. No significant difference was found in SpO_2_, ETCO_2_, PaCO_2_, Paw, HR and MAP among the three groups at each point (Table 2).

**Table 2:** The level of S_P_O_2_, ETCO_2_, PaCO_2_, PAW, HR and MAP among three groups

	Group	T_1_	T_2_	T_3_	T_4_	T_5_	T_6_
PaO_2_ (mm Hg)	P	272 ± 37^*^	153 ± 32^*#^	132 ± 29^*#^	121 ± 27^*#^	115 ± 35^*^	153 ± 45^*^
	L	268 ± 58^*^	128 ± 31^*^	106 ± 22^*^	97 ± 20^*^	112 ± 33^*^	141 ± 31^*^
	C	465 ± 58	246 ± 73	230 ± 50	185 ± 54	195 ± 42	216 ± 34
S_P_O_2_ (%)^§^	P	99.5 ± 0.7	99.1 ± 1.1	98.2 ± 3.0	98.3 ± 1.5	98.3 ± 1.5	99.4 ± 0.9
	L	99.5 ± 0.9	98.8 ± 2.0	98.2 ± 2.1	97.8 ± 2.1	98.5 ± 1.6	99.3 ± 1.2
	C	99.6 ± 0.8	99.3 ± 1.4	100.0 ± 0.0	99.9 ± 0.4	100.0 ± 0.0	100.0 ± 0.2
SvO_2_ (%)	P	75.0 ± 4.1	66.7 ± 9.5^*^	61.8 ± 11.3^*^	56.2 ± 9.0^*^	60.3 ± 12.2^*^	61.1 ± 7.3^*^
	L	74.8 ± 6.2	67.4 ± 10.5^*^	61.8 ± 13.0^*^	58.8 ± 10.7^*^	60.6 ± 9.1^*^	60.9 ± 9.1^*^
	C	75.5 ± 3.9	72.4 ± 8.5	68.3 ± 10.7	64.2 ± 11.4	66.5 ± 8.9	66.0 ± 10.7
ETCO_2_ (mmHg)	P	35.4 ± 5.9	36.8 ± 4.6	35.7 ± 4.1	34.7 ± 4.0	35.1 ± 4.4	34.4 ± 4.4
	L	35.6 ± 5.3	36.6 ± 4.2	36.0 ± 4.1	35.3 ± 3.6	35.4 ± 3.8	34.1 ± 4.0
	C	35.7 ± 55.3	36.0 ± 4.2	35.4 ± 4.4	34.9 ± 4.4	34.9 ± 4.4	35.4 ± 3.8
PaCO_2_ (mmHg)	P	42.9 ± 6.0	42.9 ± 5.9	41.5 ± 5.7	40.1 ± 5.8	41.6 ± 6.3	39.5 ± 6.7
	L	42.5 ± 4.4	41.5 ± 6.2	41.3 ± 5.3	41.3 ± 5.8	40.4 ± 6.3	39.7 ± 7.0
	C	43.1 ± 5.1	42.6 ± 6.2	42.6 ± 5.5	42.0 ± 6.2	41.3 ± 6.8	41.4 ± 5.4
PAW (cmH_2_O)	P	14.9 ± 3.2	22.5 ± 3.7	23.0 ± 3.4	23.6 ± 4.5	24.3 ± 4.5	24.3 ± 3.9
	L	14.4 ± 2.6	23.1 ± 3.9	23.2 ± 4.4	23.7 ± 3.9	24.6 ± 3.9	24.9 ± 4.5
	C	15.2 ± 3.0	22.5 ± 3.7	24.2 ± 4.7	24.1 ± 3.8	23.5 ± 3.7	24.2 ± 4.4
HR (bpm)	P	78 ± 14	77 ± 13	78 ± 16	78 ± 15	76 ± 13	71 ± 11
	L	74 ± 13	75 ± 12	77 ± 10	77 ± 11	74 ± 13	73 ± 18
	C	75 ± 13	74 ± 13	74 ± 13	76 ± 10	75 ± 13	74 ± 10
MAP (mmHg)	P	102 ± 15	96 ± 16	93 ± 16	97 ± 15	99 ± 12	96 ± 11
	L	97 ± 12	98 ± 13	93 ± 13	94 ± 13	101 ± 9	98 ± 13
	C	99 ± 14	98 ± 12	94 ± 15	101 ± 15	99 ± 10	98 ± 21

T_1_, post-anesthesia/pre-nebulization; T_2_, OLV 10 min; T_3_, OLV 15 min; T_4_, OLV 30 min; T_5_, OLV 60 min; T_6_, OLV 120 min; ^*^p<0.05 compared with group C; ^#^*p*<0.05 compared with group L; ^§^The SpO_2_ in patients with hypoxemia was significantly lower than patients in Group P and C

**Fig. 3:**
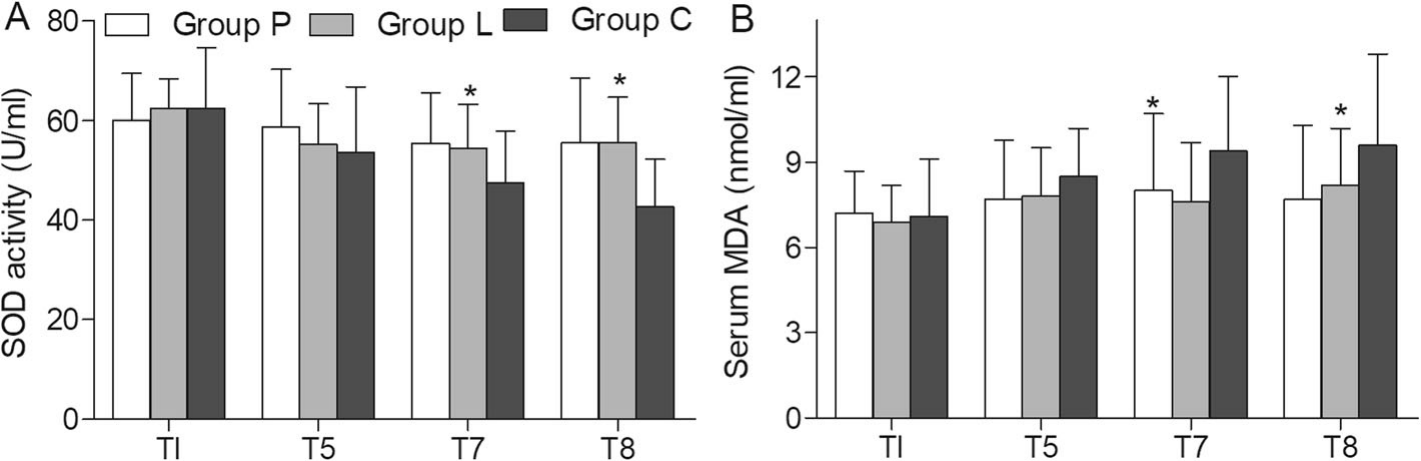
Serum SOD activities and MDA levels in three groups. Comparison of Serum SOD activities (**a**) and MDA levels (**b**) in Group P, L and C at T1 (post-anesthesia/pre-nebulization), T5 (OLV 60 min), T7 (30min after switching back to TLV), T8 (24h post-operation). *p < 0.05 vs. Group C

At 30 min after restarting TLV and 24 h after surgery, the levels of SOD in Group P and L were significantly higher than Group C, while MDA were significantly lower (Fig. 3). No significant difference was found in SOD and MDA levels between Group P and L. After the surgery, there was no significantly difference in the ICU stays and hospital stays among the three groups (*p >* 0.08). No patients had complications after surgery in Group P and L, while 2 patients had pulmonary infection, 1 patient had pulmonary infection and pulmonary atelectasis and 1 patient had liver dysfunction in Group C (Table 3). The risk of complications was significantly higher in Group C than in Group P and L (*p* = 0.012).

**Table 3:** Clinical data of patients among three groups after surgery

	Group P (*n* = 30)	Group L (*n* = 28)	Group C (*n* = 30)	P
Hospital stays (d)	20.3 ± 2.4	27.3 ± 26.3	26.0 ± 12.7	0.246
ICU stays (d)	1^a^	1.2 ± 0.6	1.0 ± 0.2	0.08
Complication^**b**^	0	0	4	0.012
Pulmonary infection	0	0	3	0.037
Pulmonary atelectasis	0	0	1	
Liver dysfunction	0	0	1	

^a^all the patients in Group P stayed 1 day

^**b**^Group P and L were combined when *Fish test* was performed

## Discussion

Although previous study had shown that PGE_1_ could decrease pulmonary shunt and increase PaO_2_ in a dose-dependent manner during OLV [12], it remains unclear whether PGE_1_ can help to maintain adequate oxygenation with a low FiO_2_ during OLV. Our article showed that low FiO_2_ (0.6) led to lower levels of PaO_2_, PaO_2_/FiO_2_, Qs/Qt and PvO_2_ than high FiO_2_ (1.0). PGE_1_ can increase the levels of PaO_2_ and PaO_2_/FiO_2_ in first 30 min during OLV. More importantly, PGE_1_ can even make patients with low FiO_2_ (0.6) have a higher level of PaO_2_/FiO_2_ than high FiO_2_ (1.0) at 2 h during OLV. Meanwhile, the low FiO_2_ (0.6) did not influence the levels of ETCO_2_, PaCO_2_, Paw, HR and MAP during OLV. In addition, low FiO_2_ (0.6) can also decrease oxidative stress and complications after surgery.

Yang M et al. showed that 58% of patients with protective strategy (FiO_2_ = 0.5, PEEP = 5cmH_2_O, V_T_ = 6 mL/kg) during OLV developed hypoxemia and needed to elevate FiO_2_ to maintain an SpO_2_ > 95% [14]. While, our result showed that only 6.7% (2/30) of patients (FiO_2_ = 0.6, PEEP = 5cmH_2_O, V_T_ = 6 ~ 8 mL/kg) developed hypoxemia and needed to elevate FiO_2_. In addition, no patients in Group P developed hypoxemia. We speculated that FiO_2_ (0.6) was the lower limit for studying the benefits of low FiO_2_.

Hypoxic pulmonary vasoconstriction (HPV) is a reflex contraction of vascular smooth muscle in the pulmonary circulation in response to low regional partial pressure of oxygen (Po_2_) [25]. Due to higher PaO2 (> 100 mmHg), HPV did not occur in the ventilated lung. The pulmonary vascular resistance (PVR) of HPV in non-ventilated lung would redistribute pulmonary blood flow to the ventilated lung. Lumb Andrew B et al. showed that PVR was determined by PvO_2_ in non-ventilated lung during OLV [25]. Lower levels of PvO_2_ in Group P and L (Fig. 2d) would lead to higher levels of PVR in non-ventilated lung, which would increase blood flow to the ventilated lung and decrease shunt fraction. As anticipated, we detected lower levels of Qs/Qt (Fig. 2b) in Group P and L. PGE_1_ could dilate pulmonary artery in ventilated lung [21], whic h could also increase blood flow to the ventilated lung and decrease shunt fraction. We also detected lower levels of Qs/Qt (Fig. 2b) in Group P. In conclusion, low FiO_2_ and PGE_1_ could increase blood flow to the ventilated lung and decrease shunt fraction during OLV.

Previous studies showed that HPV had two distinct phases [26–28]. Phase 1 began within a few seconds and was maximal at 15 min. When moderate hypoxia (Po_2_ 30 to 50 mmHg) was sustained for more than 30 to 60 min, phase 2 of HPV began and a further increase in PVR was seen, reaching a peak at 2 h. In clinical practice, PaO_2_ reached its lowest level 20 to 30 min after the start of OLV and then gradually increased during the next 1 to 2 h [29]. Here, our results showed that PaO_2_ decreased and reached nadir in the first 30 min during OLV and 2 hypoxemias occurred in Group L at 10 min and 15 min during OLV, respectively. More importantly, our results further showed that PGE_1_ delayed the nadir time of PaO_2_ to 60 min, during which PVR began the second increasing. Overall, PGE_1_ was beneficial for patients to go through hypoxic period in the first 30 min during OLV. No patients in Group P had hypoxemia during OLV, supporting the conclusion.

Grubb TL et al. showed that PGE_1_ increased the level of PaO_2_/FiO_2_ and decreased the level of Qs/Qt at 15 min during OLV. Meanwhile, the level of PaO_2_/FiO_2_ and Qs/Qt returned after PGE_1_ withdrawal [21]. Our results further showed that PGE_1_ increased the level of PaO_2_/FiO_2_ in patients with low FiO_2_ (0.6) to the level in patients with high FiO_2_ (1.0) in 60 min during OLV, indicating that PGE_1_ could maintain adequate oxygenation in patients with low FiO_2_ (0.6) in 60 min during OLV. More importantly, PGE_1_ made a higher PaO_2_/FiO_2_ in patients with low FiO_2_ (0.6) than in patients with high FiO_2_ (1.0) at 2 h during OLV, indicating that PGE_1_ was beneficial to a prolong surgery.

Ultrasonic nebulizer utilizes 50 Hz AC electricity, converts it into high frequency electricity of more than 1.45 MHz and then into same frequency sound waves (ultrasonic wave). This wave generates mechanical oscillation to the drug solution, creating aerosols of 1–5 μm diameter that are of similar diameter as alveoli. Therefore, medications can enter the alveoli with airflow [22], accumulate in the lower respiratory tract and exert a fast and localized effect with minimal systemic impact [30]. As anticipated, the three groups had similar levels of MAP and HR in our study.

Although higher levels of oxidative stress in patients with higher FiO_2_ after the surgery had been demonstrated by previous studies [14, 31, 32], the levels of oxidative stress during OLV were little studied. In our article, we studied the levels of MDA and SOD at post-anesthesia/pre-nebulization, 60 min during OLV, 30 min after restarting TLV and 24 h after surgery. Our results showed that the higher oxidative stress in patients with higher FiO_2_ occurred only after the OLV. Olivant et al. [32] showed similar levels of pro-inflammatory cytokines in the plasma during OLV. However, higher levels of pro-inflammatory cytokines were detected in lung tissue after surgery. Our results explained this paradox phenomenon.

Our previous animal study showed that FiO_2_ (0.6) decreased the levels of oxidative stress and lung injury after OLV [13]. Here, our results further showed that FiO_2_ (0.6) can decrease the levels of oxidative stress and complications in esophageal cancer patients after OLV. In this article, we not only studied the benefits of lower FiO_2_ in human, but also the benefits of PGE_1_. However, there are still some limitations in this study. Firstly, the sample size was small to study the role of PGE_1_ in some rare complications, such as ARDS. Secondly, we did not study the safe dose range of PGE_1_. Thirdly, our study subjects were limited to patients with normal pulmonary function. Even though a case report had shown that epoprostenol improve oxygenation in a patient with severe interstitial lung disease during OLV [33], more articles are needed to study the role of PGE_1_ in patients with impaired pulmonary function. Finally, whether PGE_1_ had similar effects in elderly, obesity and pediatric patients needed to study.

## Conclusions

PGE_1_ can maintain adequate oxygenation in patients with low FiO_2_ (0.6) in during OLV. Reducing FiO_2_ to 0.6 during OLV can decrease the levels of oxidative stress and complications after OLV.

## Data Availability

All data generated or analyzed during this study are included in this published article.

## Abbreviations

OLV: One-lung ventilation
PGE_1_: Prostaglandin E_1_
FiO_2_: Fraction of inhaled oxygen
SOD: Superoxide dismutase
MDA: Malondialdehyde
ARDS: Acute respiratory distress syndrome
PaO_2_: Partial pressure of arterial oxygen
BIS: Bispectral index
MAP: Mean arterial pressure
Paw: Airway pressure
HPV: Hypoxic pulmonary vasoconstriction
PVR: Pulmonary vascular resistance
